# Smooth Muscle Cells of Penis in the Rat: Noninvasive Quantification with Shear Wave Elastography

**DOI:** 10.1155/2015/595742

**Published:** 2015-10-15

**Authors:** Jia-Jie Zhang, Xiao-Hui Qiao, Feng Gao, Ming Bai, Fan Li, Lian-Fang Du, Jin-Fang Xing

**Affiliations:** Department of Medical Ultrasound, Shanghai General Hospital, Shanghai Jiao Tong University School of Medicine, Shanghai 200080, China

## Abstract

*Purpose*. Smooth muscle cells (SMCs) of cavernosum play an important role in erection. It is of great significance to quantitatively analyze the level of SMCs in penis. In this study, we investigated the feasibility of shear wave elastography (SWE) on evaluating the level of SMCs in penis quantitatively.* Materials and Methods*. Twenty healthy male rats were selected. The SWE imaging of penis was carried out and then immunohistochemistry analysis of penis was performed to analyze the expression of alpha smooth muscle actin in penis. The measurement index of SWE examination was tissue stiffness (TS). The measurement index of immunohistochemistry analysis was positive area percentage of alpha smooth muscle actin (AP).* Results*. Sixty sets of data of TS and AP were obtained. The results showed that TS was significantly correlated with AP and the correlation coefficient was −0.618 (*p* < 0.001). The result of TS had been plotted against the AP measurements. The relation between the two results has been fitted with quadric curve; the goodness-of-fit index was 0.364 (*p* < 0.001).* Conclusions*. The level of SMCs in penis was successfully quantified* in vivo* with SWE. SWE can be used clinically for evaluating the level of SMCs in penis quantitatively.

## 1. Introduction

The special structure in cavernosum is the key structure for erectile function. In cavernosum, smooth muscle cells (SMCs) account for about 40–52% of cells [1] and the level of SMCs can directly affect the erectile function. Blood flows into sinusoids during the relaxation of SMCs; this is the key process of penile erection. So the blood flow of cavernosum under the erectile condition is directly determined by the number of SMCs. The decrease of SMCs will directly lead to the erectile hypofunction. Studies have found that, with age increasing, the smooth muscle tissue will gradually atrophy, cells will decrease, fibrous tissue will proliferate, and consequently the erectile function will decline [2]. The number of SMCs of cavernosum is also closely related with the sex hormone levels. Testosterone can promote the generation of SMCs and enhance the cell vitality. Cell vitality and proliferation of SMCs decline with testosterone decrease, which can lead to their atrophy and reduction in the number and the decline of erectile function [3]. Some diseases can also lead to the change of the number of SMCs. For example, Peyronie's disease can cause abnormal fibrosis of cavernosum and the decline of SMCs-fibroblasts ratio and consequently affect the erectile function [4]. Therefore, it is of great significance to quantitatively analyze the level of SMCs in penis. At present, the level of SMCs can be quantitatively analyzed only by penis biopsy and immunohistochemistry [5, 6]. But this method is not suitable for clinical expansion because of many side effects; a new technology is in urgent need.

Shear wave elastography (SWE) is a new ultrasound technology. It uses probe to emit safe acoustic radiation force impulses which can focus consecutively on different depth of tissue to cause the tissue particles to vibrate and generate shear waves. The ultrafast imaging system can precisely measure the shear wave velocity, and then the tissue stiffness (TS) can be calculated with shear wave velocity in system and can accomplish real time imaging [7–12]. Since TS is determined by cell types and amount of tissue, theoretically, SWE can be used to analyze cell types and level. Also, SWE is noninvasive, of low cost, and easy to handle. It may become a new method to diagnose pathological changes of cavernosum tissues and take the place of biopsy.

In this study, twenty rats with different level of SMCs in penis were imaged by SWE and TS was measured. Then we analyzed the correlation of TS with the amount of SMCs and investigated the feasibility of SWE on evaluating the level of SMCs in penis quantitatively and noninvasively.

## 2. Materials and Methods

### 2.1. Animals

All animal experiments were approved by Institutional Animal Care and Use Committee. Twenty healthy male Sprague Dawley rats (1.5–14 months) obtained from the Animal Breeding Center were randomly sampled. The rats' weight was 337.12 ± 102.64 g, and length of body was 13.43 ± 4.92 cm.

### 2.2. Reagents

The anti-alpha actin antibody [SA-20] (ab 82247) and rabbit anti-rat IgG H&L (HRP) (ab 6734) were obtained from Abcam (Shanghai, China).

### 2.3. SWE Examination

All rats were anesthetized with sodium pentobarbital (30 mg/kg, ip), and anesthesia was maintained with supplemental sodium pentobarbital as needed. The penis of rat was in a flaccid state below narcotism. The ultrafast ultrasound device Aixplorer (SuperSonic Imagine, Aix-en-Provence, France) was used for SWE imaging and the probe selected was SuperLinear SL15-4. The four limbs of the rat were fixed supinely after it was anesthetized and the two-dimensional ultrasonography was performed after penis was fully exposed. The transverse section was selected for scanning. Then SWE was carried out after penis was shown clearly and the SWE imaging box should be larger than the transverse section of penis. The near glans, mid, and the near root segment of penis were, respectively, selected for SWE imaging and the images were saved in real time. The information regarding the Aixplorer settings was as follows: the focal depth was 1-2 cm, the gain was 30%, the frame for averaging was 12 Hz, and the penetration mode selection of SWE was “Pen.”

After SWE examination, the saved images were used for measurement. Q-Box was used to measure TS in SWE imaging box. The delineated standard of the Q-Box (circle) was surrounding penis on the largest scale with the capsule as the outer boundary. Therefore, the diameter of Q-Box was different for each rat; it was determined by the size of penis. TS of the near glans, mid, and near root segment of penis was, respectively, measured. The unit was kilopascal (kPa).

### 2.4. Histological Examination

Each rat was killed immediately after SWE examination. Penis was cut off at the root and fixed in 4% paraformaldehyde for 12–24 hours. After dehydration through graded concentrations of ethanol and transparence in xylene, penis was embedded in paraffin. The paraffin-embedded tissue was cut into 5 *μ*m sections and adhered to SuperFrost Plus slides. These prepared sections were used for hematoxylin-eosin (HE) and immunohistochemical staining.

The sections were stained with hematoxylin for 10 min, followed by being rinsed several times with flowing water, and then they were stained with eosin for 20–30 s. After dehydration through graded alcohol and transparence in xylene, the sections were mounted with neutral gum and baked at the temperature of 37°C for drying.

After heat drying, the sections were deparaffinized in xylene and subsequently rehydrated in gradients of ethanol. The sections were treated with 3% hydrogen peroxide to inactivate endogenous peroxidase, and then the antigen was retrieved at 95°C for 10 min. The sections were incubated with the normal goat serum at 37°C for 15 min and then primary antibody, anti-alpha actin antibody (1 : 50), was applied, and the sections were incubated at 4°C overnight. Subsequently, the sections were incubated with rabbit anti-rat secondary antibody for 15 min and then incubated with streptavidin-peroxidase complex for 15 min. Colored reactions were developed by incubating with 3′3′-diaminobenzidine and subsequently counterstained with hematoxylin. After dehydration and transparence, the sections were mounted with neutral gum. Between all reaction steps, the slides were rinsed with 0.1 M phosphate buffered saline (pH 7.4).

### 2.5. Image Collection and Analysis

The sections with HE staining were observed under light microscope and the color image analysis system was used to analyze immunohistochemical images. The image analysis and data measurement were performed by professional pathologists with more than 5 years of experiences. The positive area percentage of alpha-smooth muscle actin (AP) of the near glans, mid, and near root segment of penis was, respectively, measured by the software of Leica QWin Plus (Wetzlar, Germany).

### 2.6. Statistical Analysis

The Kolmogorov-Smirnov (K-S) test was used to determine whether the data sets (TS and AP of rats) followed normal distribution. If necessary, the nonparametric method (Spearman test) would be used to analyze the correlation of TS with AP. The statistical analyses were performed by SPSS software (v. 18.0 for Windows; SPSS Inc., Chicago, USA). A *p* value of < 0.05 was considered statistically significant.

## 3. Results

### 3.1. SWE Imaging

**Figure 1:**
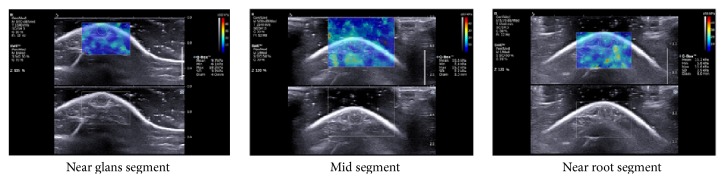
SWE imaging of penis. The two-dimensional image (lower) shows that the transverse section of penis is oval with intact capsule and uniformly hypoechoic inside. The SWE image (upper) shows that each area of penis is filled with color, the image is like an oil paint, and there are no mosaic-like points. Q-Box was a circle depicted with tunica albuginea as the boundary. (Q-Box, measurement results of TS; Min, minimum; Max, maximum; SD, standard deviation; Diam, diameter.)

All rats' penises were imaged successfully (Figure 1). The two-dimensional images showed that the transverse section of penis was oval with clear boundary and intact capsule and was uniformly hypoechoic inside. The SWE images showed that each area of penis was filled with color, the image was like an oil painting, and there were no mosaic-like points (area without SWE signal). TS of all rats was 9.10 ± 1.63 kPa.

### 3.2. HE Staining and Immunohistochemistry Analysis

**Figure 2:**
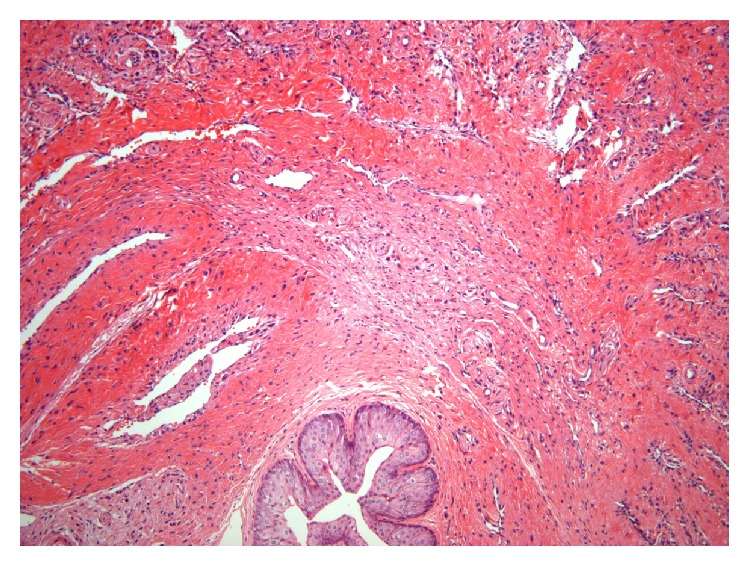
Sections of rat's penile tissue (HE 100x). The outer layer is the corpus cavernosum; the inner layer is the corpus spongiosum. At the center of the corpus spongiosum is the urethra (cavity) which is covered with urethral epitheliums (blue staining). The penile tissue is mainly composed of SMCs and fibroblasts and the arrangement.

**Figure 3:**
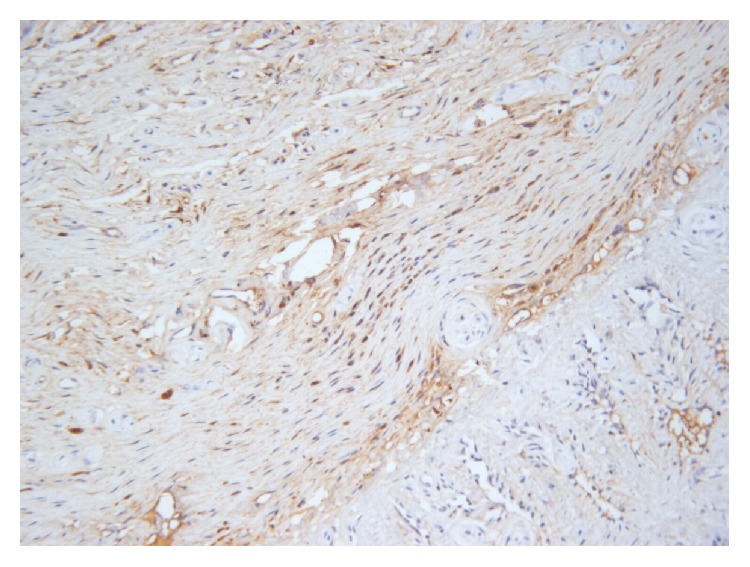
The expression of alpha-actin in rats' penile tissue. The brown color shows the positive cell in this area. In this figure, the positive cells are smooth muscle cells (400x).

The rats' penile tissue mainly included SMCs and fibroblasts (Figure 2). The sections with immunohistochemical staining were obtained successfully.  Figure 3 shows the expression of alpha-actin of penis. AP of all rats was 10.55 ± 9.13%, AP of the near glans segment of all rats was 10.60 ± 8.93%, AP of the mid segment was 10.77 ± 10.02%, and AP of near the root segment was 10.27 ± 8.87%.

### 3.3. Correlation Analysis

**Figure 4:**
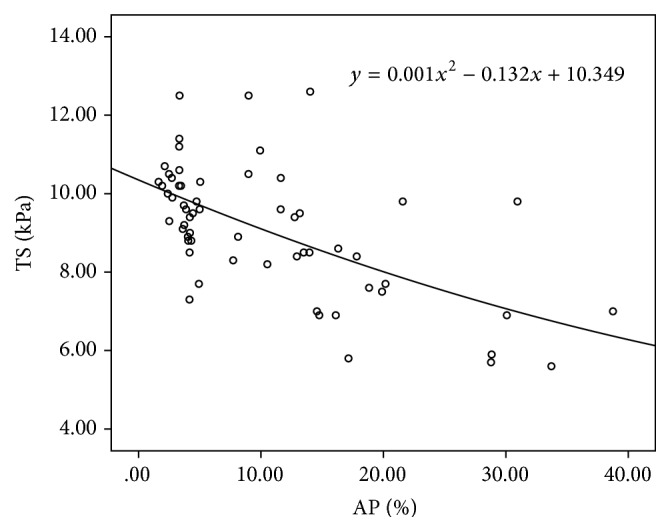
The result of TS plotted against the AP measurements of each rat. The relation between the two results has been fitted with quadric curve.

For each rat, the near glans, mid, and near root segment of penis were, respectively, selected for the measurement of TS and histological examinations. Sixty sets of data of TS and AP were obtained. We found that the data of TS followed normal distribution (*p* = 0.909), but the data of AP did not. So the Spearman test was used to analyze the correlation between TS and AP. The result showed that TS was significantly correlated with AP (*p* < 0.001); the correlation coefficient was −0.618. The result of TS has been plotted against the AP measurements of each rat (Figure 4). The relation between the two results has been fitted with quadric curve (*y* = 0.001*x* ^2^ − 0.132*x* + 10.349); the goodness-of-fit index was 0.364 (*p* < 0.001).

## 4. Discussion

Penis is mainly composed of corpora cavernosa and corpus spongiosum, and the cell types include SMCs and fibroblasts. SMCs are the structural basis of cavernosum relaxation and account for about 40–52% in cavernosum. The main process of penile erection is as follows: with sexual stimulation, SMCs relax and then corpora cavernosum penis congests and swells followed by the gradual increase of intracavernous pressure. At the same time, the subalbugineous veniplex is compressed which can lead to the decrease of blood reflux. When the intracavernous pressure is high enough, the inflow of penis arteries stops and there is no outflow. Thus the penis is under a fully closed condition and achieves full erection [13, 14]. Meanwhile, the effective venoocclusion is directly affected by the SMCs-fibroblasts ratio. Therefore, the level of SMCs can directly impact the erectile function.

So far, the only way to evaluate the level of SMCs in penis is obtaining samples by penis biopsy and quantifying cell amount by immunohistochemistry. The mechanism of quantifying the amount of SMCs by immunohistochemistry is as follows: SMCs in penis include cavernosum SMCs and vascular SMCs, mainly cavernosum SMCs [1], which have the function of contraction and contain alpha-actin. And anti-alpha actin antibody can serve as the marker of SMCs. But this method needs high technical conditions when drawing materials and is invasive and liable to cause some severe side effects including penis pain and penile induration. Therefore, it is difficult to be accepted by patients and the clinical application has been greatly restricted. It is necessary to explore a noninvasive method that can be applied on clinical evaluation of the level of SMCs in penis.

SWE is a new technology which can be used to analyze TS clinically. Since TS is closely associated with cell types and level of tissue, SWE can be used to analyze cell types and level. SWE uses the ultrafast imaging system to precisely record the tissue movement induced by the propagation of shear waves and the velocity of the tissue movement is quantified using tissue Doppler techniques; thus the propagation of shear waves through the scanning plane can be monitored in real time. The shear wave velocity getting through each particle of tissue is estimated using cross correlation algorithms. Then the TS can be calculated with shear wave velocity in system. At the same time, the TS can be color coded and superimposed on a B-mode image, and then a real time TS map of* in vivo* tissue is formed [1, 11, 12]. Since SWE is hopeful to be a new method for analyzing types and level of cells, we attempted to analyze the level of SMCs in penis with SWE.

In this study, anti-alpha actin antibody was selected to quantitatively measure the amount of SMCs and the measurement index was AP. Then the correlation of TS with AP was analyzed. The result showed that TS was significantly negatively correlated with AP of all rats. It suggests that there is a significant negative correlation of TS with SMCs in penis. This finding just fits with the common understanding that more SMCs in penis means better elasticity and lower TS of penis. At the same time, we got the quantitative relationship between AP and TS of penis for the first time. The curve-fitting equation was TS = 0.001AP^2^ − 0.132AP + 10.349. Therefore, SWE is promising to be a new noninvasive method of evaluating the level of SMCs and it deserves further study.

The transverse section was used for SWE measurements in this study as we considered the anisotropy of SMCs. Based on the anisotropy of SMCs, the shear wave speed along the fiber could be significantly higher than that across the fiber. When SWE imaging was carried out, shear waves would propagate along the fiber if the section was parallel to the fiber, and shear waves would travel across the fiber if the section was perpendicular (or nearly perpendicular) to the fiber. Therefore, when the longitudinal section was used for SWE imaging, the influence of the anisotropy of the SMCs for shear wave velocity could not be avoided because it was impossible to ensure that each section was parallel to the fiber in practice. When the transverse section was selected, the influence of the anisotropy of the SMCs for shear wave velocity could be overcome effectively because it was possible to ensure that each section was perpendicular (or nearly perpendicular) to all fibers.

There are some limitations in this study. We could not identify the corpus spongiosum and the corpus cavernosum of rats in the two-dimensional ultrasonography, so we measured the TS of the whole penis. But the cell types of the corpus spongiosum are close to the corpus cavernosum, mainly including SMCs and fibroblasts, so our result is also suitable for the corpus cavernosum. Furthermore, since the corpus spongiosum and the corpus cavernosum of human beings can be clearly divided in the two-dimensional ultrasonography, it would not happen when this technology is used clinically.

## 5. Conclusion

In our research, the level of SMCs in penis was successfully quantified* in vivo* with SWE. SWE can be used clinically for evaluating the level of SMCs in penis quantitatively, and it deserves further study.
